# A simple way to measure the burden of interval cancers in breast cancer screening

**DOI:** 10.1186/1471-2407-14-782

**Published:** 2014-10-24

**Authors:** Sune Bangsbøll Andersen, Sven Törnberg, Elsebeth Lynge, My Von Euler-Chelpin, Sisse Helle Njor

**Affiliations:** Department of Public Health, University of Copenhagen, CSS, Øster Farimagsgade 5, 1014 Copenhagen K, Denmark; Department of Cancer Screening, Regional Cancer Centre and Karolinska Institutet, Hälso- och Sjukvårdsförvaltningen, Regionalt cancercentrum, Box 6909, 102 39 Stockholm, Sweden; Department of Public Health, Centre for Epidemiology and Screening, University of Copenhagen, Øster Farimagsgade 5, opg. B, Postboks 2099, DK-1014 Copenhagen K, Denmark

**Keywords:** Mammography, Screening, Interval cancer, Program evaluation, Sensitivity, Quality measure, Background incidence

## Abstract

**Background:**

The sensitivity of a mammography program is normally evaluated by comparing the interval cancer rate to the expected breast cancer incidence without screening, i.e. the proportional interval cancer rate (PICR). The expected breast cancer incidence in absence of screening is, however, difficult to estimate when a program has been running for some time. As an alternative to the PICR we propose the interval cancer ratio . We validated this simple measure by comparing it with the traditionally used PICR.

**Method:**

We undertook a systematic review and included studies: 1) covering a service screening program, 2) women aged 50-69 years, 3) observed data, 4) interval cancers, women screened, or interval cancer rate, screen detected cases, or screen detection rate, and 5) estimated breast cancer incidence rate of background population. This resulted in 5 papers describing 12 mammography screening programs.

**Results:**

Covering initial screens only, the ICR varied from 0.10 to 0.28 while the PICR varied from 0.22 to 0.51. For subsequent screens only, the ICR varied from 0.22 to 0.37 and the PICR from 0.28 to 0.51. There was a strong positive correlation between the ICR and the PICR for initial screens (r = 0.81), but less so for subsequent screens (r = 0.65).

**Conclusion:**

This alternate measure seems to capture the burden of interval cancers just as well as the traditional PICR, without need for the increasingly difficult estimation of background incidence, making it a more accessible tool when evaluating mammography screening program performance.

**Electronic supplementary material:**

The online version of this article (doi:10.1186/1471-2407-14-782) contains supplementary material, which is available to authorized users.

## Background

Mammography screening is intended to reduce breast cancer mortality by detecting the breast cancer cases at an earlier stage. A high sensitivity is needed for a mammography screening program to fulfil its purpose. This means the program should not have too many interval cancers, i.e. cancers that appear clinically after a negative screening result and before the next scheduled screen. A screening program in a population with a high breast cancer incidence can have a high interval cancer rate and still have as protective an effect on breast cancer mortality as a screening program with a low interval cancer rate running in a population with a low breast cancer incidence. The sensitivity of a mammography screening program is therefore normally evaluated by comparing the interval cancer rate to the expected breast cancer incidence without screening, i.e. the PICR [1]. In order to compare sensitivity across screening programs, the European guidelines provide acceptable and desirable values for this measure. However, over time the difficulties in estimating the expected background incidence makes such comparisons increasingly unreliable.

The expected breast cancer incidence in absence of screening, or background incidence, is difficult to approximate, as the introduction of a screening program makes it difficult to find an unscreened, comparable population group. As the breast cancer incidence has changed over time [2], it will, some years after introducing of screening, no longer be meaningful to estimate the expected breast cancer incidence without screening from the breast cancer incidence prior to the screening.

The aim of this article is to propose and validate an alternative performance indicator for the burden of interval cancers in an organized mammography screening program. We aim to validate this proposed measure by comparing with the PICR from studies of service screening programs for women aged 50-69. Zorzi et al. [3] have previously proposed that for a given subsequent screening round, PICR is substituted by . We propose to use the even simpler  and to use this measure also for the initial screening round.

## Methods

### Search strategy

**Figure 1:**
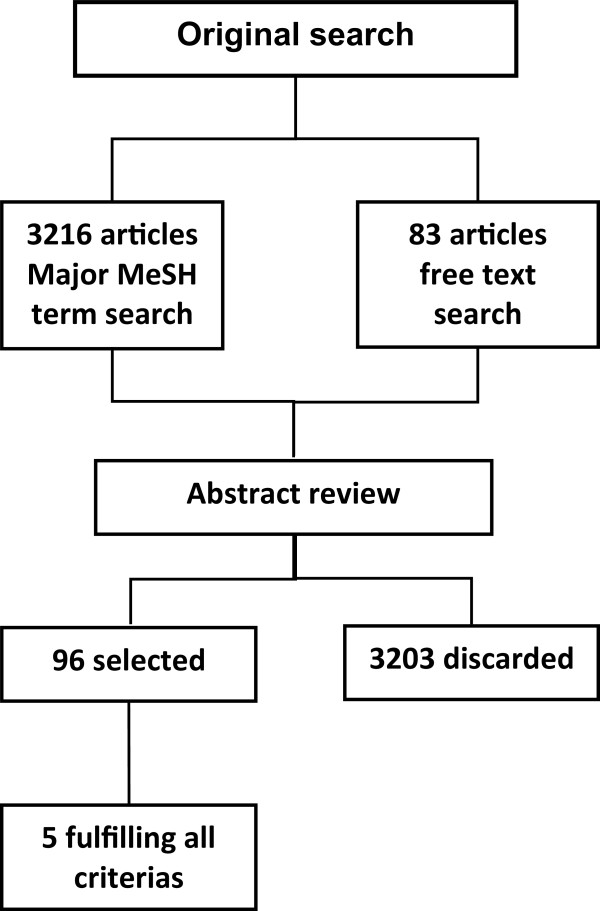
**Flow diagram of selection of papers.**

We performed a PubMed search using Major MeSH terms with the restriction of the words “mammography” or “screening” required in the abstracts where abstracts were available, in the title where abstracts were not available, and finally in free texts, see Additional file 1. We did this search in March 2012, and it was limited to publications in English. This search resulted in 3299 matches. Among these matches, relevant studies were identified in a two-step search. First, two independent researchers, SBA & SHN, reviewed the titles and abstracts of the 3299 papers. This sorting resulted in 96 papers for further consideration. Second, we selected studies: 1) covering a service screening program, 2) including women aged 50-69 years, 3) reporting observed data (paper based on modeling only were excluded), 4) reporting number of screen detected cancers or screen detection rates and number of screened women and two of these: number of interval cancers, interval cancer rate or number of screened women and 5) reporting estimated breast cancer incidence rate of the background population in the absence of screening. Third, in case consensus was not obtained, a third researcher, EL, participated in the decision. This resulted in inclusion of 5 papers [4–8] describing 12 different screening programs, to be included in this review, Figure 1.

### Definitions

#### Screen detected cancers

A primary breast cancer found at scheduled screening examination. Some centers allowed a so-called early recall (or intermediate mammography) prescribed for diagnostic reasons 1 year after the screening test. Cases detected at early recall are calculated as SD cancers.

#### Interval cancer

A primary breast cancer diagnosed in a woman, after a screening test negative for malignancy. The breast cancer should either be diagnosed before the next invitation to screening, or within a time period equal to the screening interval in case the woman has reached the upper age limit for screening or for other reasons does not receive more invitations.

#### Proportional interval cancer rate (PICR)

Interval cancer rate as a proportion of the underlying, expected, breast cancer incidence rate in the absence of screening: . This is the classic epidemiology performance indicator [9] as used in the EU Guidelines [1].

#### Interval cancer ratio (ICR)

Interval cancer as proportion all cancers: . This is the measure we propose as an alternative performance indicator.

### Data extraction

From each paper we extracted: Information on number of screened women and number of screen detected cancers or screen detection rate, the expected background annual incidence rate per 10,000 and number of interval cancer cases. If not provided, we calculated interval cancer cases per 10,000 screen negative women (this being number of women screened minus number of screen detected cases). Finally we calculated  and . In the Veneto region study the interval cancers were identified by linkage to the regional hospital discharge records. For all other studies interval cancers were identified by linkage to the regional/national cancer register, which all are regarded as complete.

### Initial versus subsequent screens

The number of screen detected cases is higher in initial screens than in subsequent screens. Therefore the ICR will be lower in initial screens than in subsequent screens. When comparing interval cancer ratios one therefore has to distinguish between initial screens and subsequent screens. All studies had a screening interval of 2 years, except Marseille where the screening interval was 3 years.

### Analysis

Pearson’s correlation coefficient and best-fit straight line was calculated using Microsoft Office Excel 2007.

## Results

The ICR in studies of initial screens varied from 0.10 to 0.28 while the PICR varied from 0.22 to 0.51 in the same studies (Table 1). In studies of subsequent screens the ICR varied from 0.22 to 0.37 with the PICR varying from 0.28 to 0.61 (Table 2). Four studies reported on mixed initial and subsequent screens. The Italian study from the Veneto Region with a majority of initial screens, had an ICR of 0.18, and a PICR of 0.29. The studies from Copenhagen, Denmark, Funen, Denmark and Pirkanmaa, Finland with a majority of subsequent screens, had an ICR of 0.25-0.34 and a PICR of 0.40-0.61.

**Table 1:** **Screened women, screen detected cancers, interval cancers and background annual incidence by screening location in primarily initial screening rounds**

Reference	Screening program location	Year (of invitation)	Age	Screened women	Screen-detected cases	Interval cancers	Pct. of initial screens	[Screen-detected per 10.000]	[Interval cancers per 10.000]	Background annual incidence rate per 10.000		
Mammography screening evaluation group [5]	Copenhagen, Denmark	1991-‘93	50-69	30,362	360	52	100	118.6	17.3	25.4	0.34	0.13 (0.10-0.16)
Njor et al. [6]	Funen, Denmark	1993-‘95	50-69	41,480	398	87	100	95.9	21.2	24.2	0.43	0.18 (0.15-0.21)
Törnberg et al. [7]	Stockholm, Sweden	1989-‘97	50-69	188,032	1,108	382	100	58.9	20.4	25.8	0.40	0.26 (0.24-0.28)
Törnberg et al. [7]	Four counties, Norway	1996-‘97	50-69	126,779	852	207	100	67.2	16.4	20.0	0.41	0.20 (0.18-0.22)
Hofvind et al. [4]	NBCSP, Norway	1996-‘05	50-69	367,428^a^	2,351	669	100	64.0	18.3	18.0	0.51	0.22 (0.21-0.23)
Törnberg et al. [7]	Marseille, France	1993-‘98	50-69	103,946	483	179	100	46.5	17.3	20.1	0.43	0.27 (0.24-0.30)
Törnberg et al. [7]	Strasbourg, France	1989-‘97	50-65	63,235	328	129	100	51.9	20.5	22.6	0.45	0.28 (0.24-0.32)
Törnberg et al. [7]	Florence, Italy	1990-‘94	50-69	35,754	325	47	100	90.9	13.3	22.2	0.30	0.13 (0.10-0.16)
Törnberg et al. [7]	Turin, Italy	1992-‘96	50-69^b^	28,804	248	40	100	86.1	14.0	20.2^c^	0.35	0.14 (0.10-0.18)
Vettorazzi et al. [8]	Veneto Region, Italy	1999-‘02	50-69	94,874^d^	683	154	73	72.0	16.3	27.8	0.29	0.18 (0.15-0.21)
Törnberg et al. [7]	Navarra, Spain	1990-‘96	45-65	40,665	256	29	100	63.0	7.2	16.2	0.22	0.10 (0.07-0.13)

^a^Only 367,428 prevalent screens from a total of 467,343 women had 2 years of follow-up.

^b^Although the age group targeted in Turin is 50-69 years, during the period of the study, invitations were restricted to women aged 50-59. A few women had the test shortly after they turned 60.

^c^Based on the ages 50-64.

^d^Women-Years at risk. Follow-up was not complete in the second year of the interval resulting in only 77,979 women-years.

**Table 2:** **Screened women, screen detected cancers, interval cancers and background annual incidence by screening location in primarily subsequent screening rounds**

Reference	Screening program location	Year (of invitation)	Age	Screened women	Screen-detected cases	Interval cancers	Pct. of initial screens	[Screen-detected per 10.000]	[Interval cancers per 10.000]	Background annual incidence rate per 10.000		
Mammography screening evaluation group [5]	Copenhagen, Denmark	1993-‘95	50-69	26,063	163	53	18	62.5	20.5	25.4	0.40	0.25 (0.19-0.31)
Njor et al. [6]	Funen, Denmark	1996-‘97	50-69	43,543	227	105	19	52.1	24.2	26.0	0.47	0.32 (0.27-0.37)
Törnberg et al.[7]	Stockholm, Sweden	1989-‘97	50-69	270,260	1,075	584	0	39.8	21.7	23.7	0.46	0.35 (0.33-0.37)
Hofvind et al.[4]	NBCSP, Norway	1998-‘05	50-69	336,323^a^	1,648	610	0	49.0	18.2	18.2	0.51	0.27 (0.25-0.29)
Törnberg et al.[7]	Pirkanmaa, Finland	1988-‘97	50-69	75,927	235	121	42	31.0	16.0	13.1^b^	0.61	0.34 (0.29-0.39)
Törnberg et al.[7]	Marseille, France	1993-‘98	50-69	36,140	171	65	0	47.3	18.1	20.1	0.45	0.28 (0.22-0.34)
Törnberg et al.[7]	Strasbourg, France	1989-‘97	50-65	104,951	390	230	0	37.2	22.0	22.6	0.49	0.37 (0.33-0.41)
Törnberg et al.[7]	Florence, Italy	1990-‘94	50-69	13,394	54	28	0	40.3	21.0	22.2	0.47	0.34 (0.24-0.44)
Törnberg et al.[7]	Turin, Italy	1992-‘96	50-69^c^	13,117	82	25	0	62.5	19.2	20.2^d^	0.47	0.23 (0.15-0.31)
Törnberg et al.[7]	Navarra, Spain	1990-‘96	45-65	85,653	268	77	0	31.3	9.0	16.2	0.28	0.22 (0.18-0.26)

^a^Only 336,323 prevalent screens from a total of 467,343 women had 2 years of follow-up.

^b^based on the ages 50-59 years.

^c^Although the age group targeted in Turin is 50-69 years, during the period of the study, invitations were restricted to women aged 50-59. A few women had the test shortly after they turned 60. and all women invited for the first time in their 50s received their subsequent invitations even after they turned 60.

^d^Based on the ages 50-64.

All studies estimated the expected background incidence by the observed incidence just before the mammography screening program started. With the breast cancer incidence increasing over time [2], this estimated background incidence will consequently increasingly underestimate the true background incidence.

The Norwegian NBCSP study estimated the background incidence by the observed incidence in women aged 50-69 years before screening started. This will underestimate the expected incidence, since the observed interval cancer rate will derive from women on average being two years older.

The Italian Veneto Region study is based on invasive cancers only, whereas all other studies are based on invasive + ductal carcinoma in situ (DCIS). Since DCIS is far more common among screen-detected cancers calculations excluding DCIS will increase the ICR more than the PICR.

**Figure 2:**
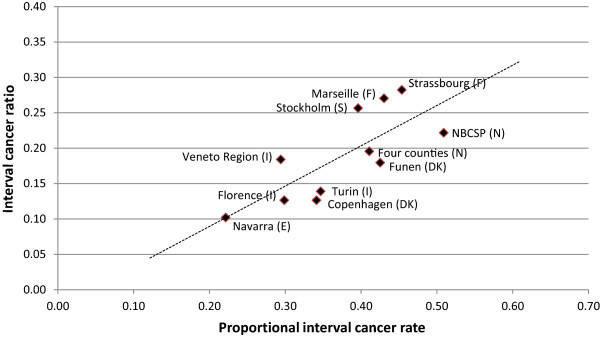
**Relationship between total IC-rate/BG-rate (PICR) and number of IC/number of total cancers (ICR), primarily** ***initial*** **screens.** NB. Veneto Region is the only program with mixed initial and subsequent screens. The diagonal line is the best-fit line for the observations.

**Figure 3:**
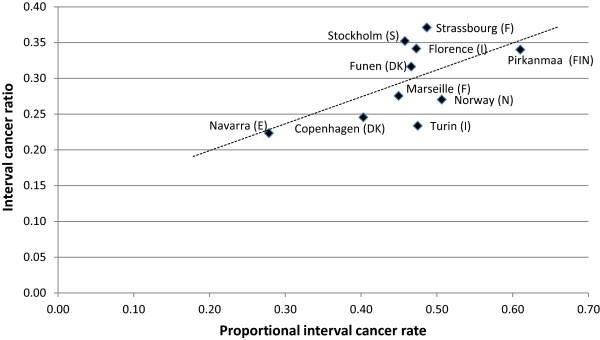
**Relationship between total IC-rate/BG-rate (PICR) and number of IC/number of total cancers (ICR), primarily** ***subsequent*** **screens.** NB. Pirkanmaa is the only program with mixed subsequent and initial screens.

The correlation between ICR and PICR was r = 0.76 for initial screens (Figure 2), and r = 0.58 for subsequent screens (Figure 3).

When comparing PICRs across screening programs, differences can reflect true differences in interval cancer rates; differences in methods for estimating the expected background incidence; or differences in the time trend of breast cancer incidence. By using the ICR, instead of estimating the PICR, the uncertainty introduced by estimating the expected background incidence is avoided. Hence, the ICR is potentially a better performance indicator as no estimation is needed. The question is, however, whether this suggested simple performance indicator captures interval cancer burden as well as the old measure.

As seen in Figure 2 there is a high positive correlation (r = 0.76) between the two measures in initial screens. Outliers are Stockholm, Norway, Copenhagen, Marseille, Strasbourg and the Italian Veneto Region. Stockholm and Norway had quite extensive opportunistic screening before the service screening program started [7, 10]. One could therefore argue that the data from these locations did not represent 100% initial screens but were probably more in line with the Veneto Region program, which had 73% initial screens. Since the ICR will be higher for subsequent screens, it was not surprising that the Stockholm, Norway and Veneto Region programs had relatively high ICR for initial screens. The high ICR for the Veneto Region was also a consequence of including only invasive cancers.

The relationship between the ICR and PICR for studies with primarily subsequent screens (seen in Figure 3) showed a strong positive correlation (r = 0.58). Data from Turin and Florence are based on small numbers (25 and 28 interval cancers respectively), and excluding these two programs gave a stronger correlation (r = 0.68).

## Discussion

When the expected background incidence is calculated based on the incidence of the general population, the actually screened population could have a different expected background incidence; especially if the attendance rate is low. Marseille had an attendance rate of 43% and had a 3 year screening interval until 2001. Strasbourg had no active invitation for the first screen, implying that the incidence of the screened population could be different from that of the general population. If we excluded Marseille and Strasbourg from the comparison, we got a correlation of r = 0.73 for initial screens. If we for subsequent screens excluded Turin, Florence, Marseille and Strasbourg we got a correlation of r = 0.73.

In randomized controlled studies (RCTs) the expected background incidence is the incidence found in the control group. PICR can therefore be calculated with great confidence in RCTs. We found information on interval cancers and screen detected cancers in both arms of the Gothenburg Breast Screening Trial [11] and the Swedish two-county trial [12]. We could only find information on number of person years and thereby incidence in the entire period wherefore the incidence in the control arm included one screening. We did neither find information stratified into initial and subsequent screenings. The value of ICR and PICR are therefore not entirely comparable with the values in the studies included in this review. Based on the results from Gothenburg Breast Screening Trial we calculated ICR = 0.21 and PICR = 0.20. From the results in the two-county trial we calculated ICR = 0.27 and PICR = 0.21. Although the results are not completely comparable the ICR and PICR values from these two RCTs are very close to the line showing the connection between ICR and PICR for subsequent screenings.

The measure we propose will make it easier to compare interval cancer rates across screening programs, since an estimation of an expected background incidence is not needed. Especially when controlling for other differences between the programs, we see a high correlation between the PICR and the ICR. It is therefore possible to get a reasonable comparison of the burden of interval cancers across mammography screening programs by comparing the ICR instead of the PICR. It does, of course, not explain other, more in-depth, issues concerning the burden of interval cancers such as difference in tumor size or stage between screen detected and interval cancers.

### Strengths & weaknesses

This study includes data from many mammography screening programs throughout Western Europe, which support the potential for use of this simple measure in different settings. As pointed out by the very limited number of studies available for this study, only a few programs actually estimate PICR and thereby check if the sensitivity follows the European guidelines. It is much simpler to calculate ICR, and we therefore believe that reporting of the program sensitivity would be much more common if the gold standard was to use ICR. Using the ICR as a performance indicator instead of the PICR will facilitate comparisons between screening programs.

Some of the centers included in this study allow for early recall. We adopted the method from Törnberg et al. 2010 and calculated cases detected at early recall as screen detected cancers. Whether cases detected at early recall are counted as screen detected cancers or interval cancers, will have a very minor impact on our study as we are comparing PICR = IC/(expected background incidence) to ICR = IC/(IC + SD), which is equivalent to comparing 1/(expected background incidence) to 1/(IC + SD).

It is a strength that the ICR is not affected by uncertainties in the estimates of background incidence, and the ICR is therefore not subject to over-estimation of the burden of interval cancers caused by an under-estimated background incidence. It is, however, a weakness that, unlike for the PICR, the ICR is affected by overdiagnosis, since overdiagnosis will increase the number of screen-detected cases. As the number of screen detected breast cancers is included in the denominator in the calculation of the ICR, this measure could be sensitive to overdiagnosis at screening. Reliable data on overdiagnosis have been reported from the programmes in Denmark and Florence, finding overdiagnosis to account for 1-5% of all incident breast cancers [13, 14]. Larger estimates of overdiagnosis have been reported in the literature, but they mainly reflect that the estimates are not adequately adjusted [15]. An overdiagnosis of 1-5% would change the size of ICR only marginally, wherefore it would not be a major concern in the interpretation of ICRs. Comparing programs with huge differences in overdiagnosis will still favor the program with many overdiagnosed cases. It is a trade-off when choosing one measure instead of the other, but we argue that there are fewer uncertainties involved in calculating the ICR than in calculating the PICR.

## Conclusion

In this study we proposed and validated the ICR as an alternative measure for the burden of interval cancers. The proposed measure seems to capture the burden of interval cancers just as well or better than the traditional PICR, as there is no need for estimations of background incidence. In order to further validate this proposed measure, more studies are needed. It should be noted that the measure of ICR should be seen in the context of other short-term performance indicators, and hence should not stand alone in the evaluation of screening performance.

## Electronic supplementary material

Additional file 1: **Search strategy.** (DOCX 13 KB)
