# Supplementary material for: A simple way to measure the burden of interval cancers in breast cancer screening
Source: BMC Cancer. 2014 Oct 24;14:782. doi: 10.1186/1471-2407-14-782 (PMC4219107; doi:10.1186/1471-2407-14-782)
Supplement: Supplementary file 1 — Additional file 1: Search strategy. (DOCX 13 KB) [file 12885_2014_4961_MOESM1_ESM.docx]

**Additional file 1: Search strategy**

......

(mammography[Title/Abstract] OR screening[Title/Abstract]) AND ((interval OR intervals OR frequency) AND (("Mammography"[Majr] OR "Breast Neoplasms/radiography"[Majr:noexp]) OR (("Breast Neoplasms"[Majr:noexp] OR "Carcinoma, Ductal, Breast"[Majr]) AND ((("Health Surveys/methods"[Majr] OR "Health Surveys/standards"[Majr] OR "Mass Screening/methods"[Majr] OR "Mass Screening/organization and administration"[Majr] OR "Mass Screening/standards"[Majr] OR "Mass Screening/statistics and numerical data"[Majr] OR "Mass Screening/trends"[Majr] OR "Mass Screening/utilization"[Majr]) OR ("Early Detection of Cancer/methods"[Majr] OR "Early Detection of Cancer/standards"[Majr] OR "Early Detection of Cancer/statistics and numerical data"[Majr] OR "Early Detection of Cancer/trends"[Majr] OR "Early Detection of Cancer/utilization"[Majr]) OR ("Diagnosis/standards"[Majr:noexp] OR "Diagnosis/statistics and numerical data"[Majr:noexp] OR "Diagnosis/trends"[Majr:noexp] OR "Diagnosis/utilization"[Majr:noexp])) OR ("Radiography"[Majr] AND ("Breast Neoplasms/diagnosis"[Majr:noexp] OR "Carcinoma, Ductal, Breast/diagnosis"[Majr])))) OR (("Guidelines as Topic"[Majr] OR "Health Planning Guidelines"[Majr] OR "Practice Guidelines as Topic"[Majr] OR "Guideline"[Publication Type] OR "Guideline Adherence"[Majr] OR "Health Systems Plans"[Majr] OR "Clinical Protocols"[Majr] OR "Time Factors"[Majr]) AND ("Mammography"[Majr] OR "Breast Neoplasms/radiography"[Majr:noexp] OR "Breast Neoplasms/diagnosis"[Majr:noexp] OR "Carcinoma, Ductal, Breast/diagnosis"[Majr]))))

The search strategy retrieved a total of 3216 papers. Inspection of titles and abstracts were reviewed to isolate relevant papers.

Furthermore we did a free text search on PubMed to find the newest papers:

(mammography [Title/Abstract] AND (interval[All Fields] OR intervals[All Fields] OR "frequency"[All Fields])) NOT medline[sb]

This resulted in 83 papers.

Finally we exhausted the references by reviewing the references to the papers we found relevant.

The search strategy retrieved a total of 96 papers. Inspection of these papers was reviewed to isolate relevant papers.
